# Supplementary material for: Causal Relationship between Aging and Anorexia Nervosa: A White-Matter-Microstructure-Mediated Mendelian Randomization Analysis
Source: Biomedicines. 2024 Aug 16;12(8):1874. doi: 10.3390/biomedicines12081874 (PMC11351342; doi:10.3390/biomedicines12081874)
Supplement: Supplementary file 1 [file biomedicines-12-01874-s001.zip › Supplementary Figure/Figure S4/Singe SNP plot of MR analysis for TL to ubm-b-1915.pdf]

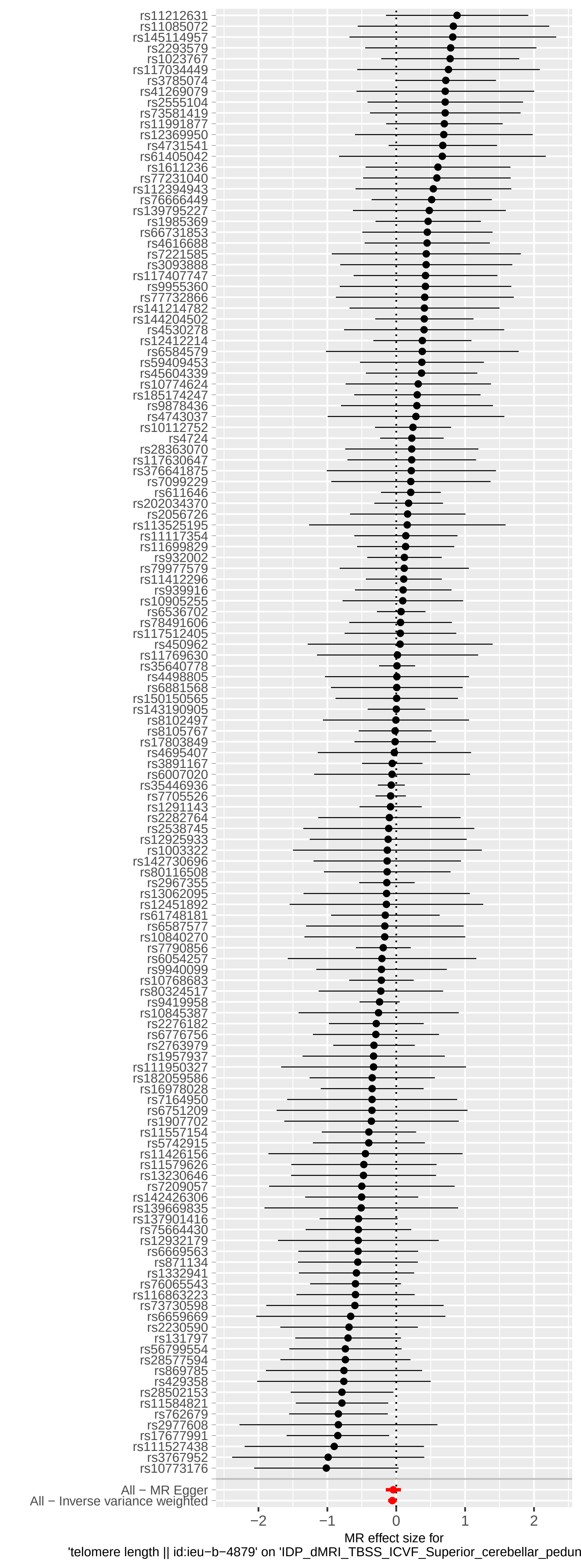
rs11212631
rs11085072
rs145114957
rs2293579
rs1023767
rs117034449
rs3785074
rs41269079
rs2555104
rs73581419
rs11991877
rs12369950
rs4731541
rs61405042
rs1611236
rs77231040
rs112394943
rs76666449
rs139795227
rs1985369
rs66731853
rs4616688
rs7221585
rs3093888
rs117407747
rs9955360
rs77732866
rs141214782
rs144204502
rs4530278
rs12412214
rs6584579
rs59409453
rs45604339
rs10774624
rs185174247
rs9878436
rs4743037
rs10112752
rs4724
rs28363070
rs117630647
rs376641875
rs7099229
rs611646
rs202034370
rs2056726
rs113525195
rs11117354
rs11699829
rs932002
rs79977579
rs11412296
rs939916
rs10905255
rs6536702
rs78491606
rs117512405
rs450962
rs11769630
rs35640778
rs4498805
rs6881568
rs150150565
rs143190905
rs8102497
rs8105767
rs17803849
rs4695407
rs3891167
rs6007020
rs35446936
rs7705526
rs1291143
rs2282764
rs2538745
rs12925933
rs1003322
rs142730696
rs80116508
rs2967355
rs13062095
rs12451892
rs61748181
rs6587577
rs10840270
rs7790856
rs6054257
rs9940099
rs10768683
rs80324517
rs9419958
rs10845387
rs2276182
rs6776756
rs2763979
rs1957937
rs111950327
rs182059586
rs16978028
rs7164950
rs6751209
rs1907702
rs11557154
rs5742915
rs11426156
rs11579626
rs13230646
rs7209057
rs142426306
rs139669835
rs137901416
rs75664430
rs12932179
rs6669563
rs871134
rs1332941
rs76065543
rs116863223
rs73730598
rs6659669
rs2230590
rs131797
rs56799554
rs28577594
rs869785
rs429358
rs28502153
rs11584821
rs762679
rs2977608
rs17677991
rs111527438
rs3767952
rs10773176
All − MR Egger
All − Inverse variance weighted
−2
−1
0
1
2
MR effect size for
'telomere length || id:ieu−b−4879' on 'IDP_dMRI_TBSS_ICVF_Superior_cerebellar_pedun
